# Supplementary figures and images for: Metastatic small cell lung cancer arises from TP53/RB1-deficient and MYC overproduction hESC-derived PNECs
Source: eLife. 2025 Jul 22;13:RP93170. doi: 10.7554/eLife.93170 (PMC12283072; doi:10.7554/eLife.93170)

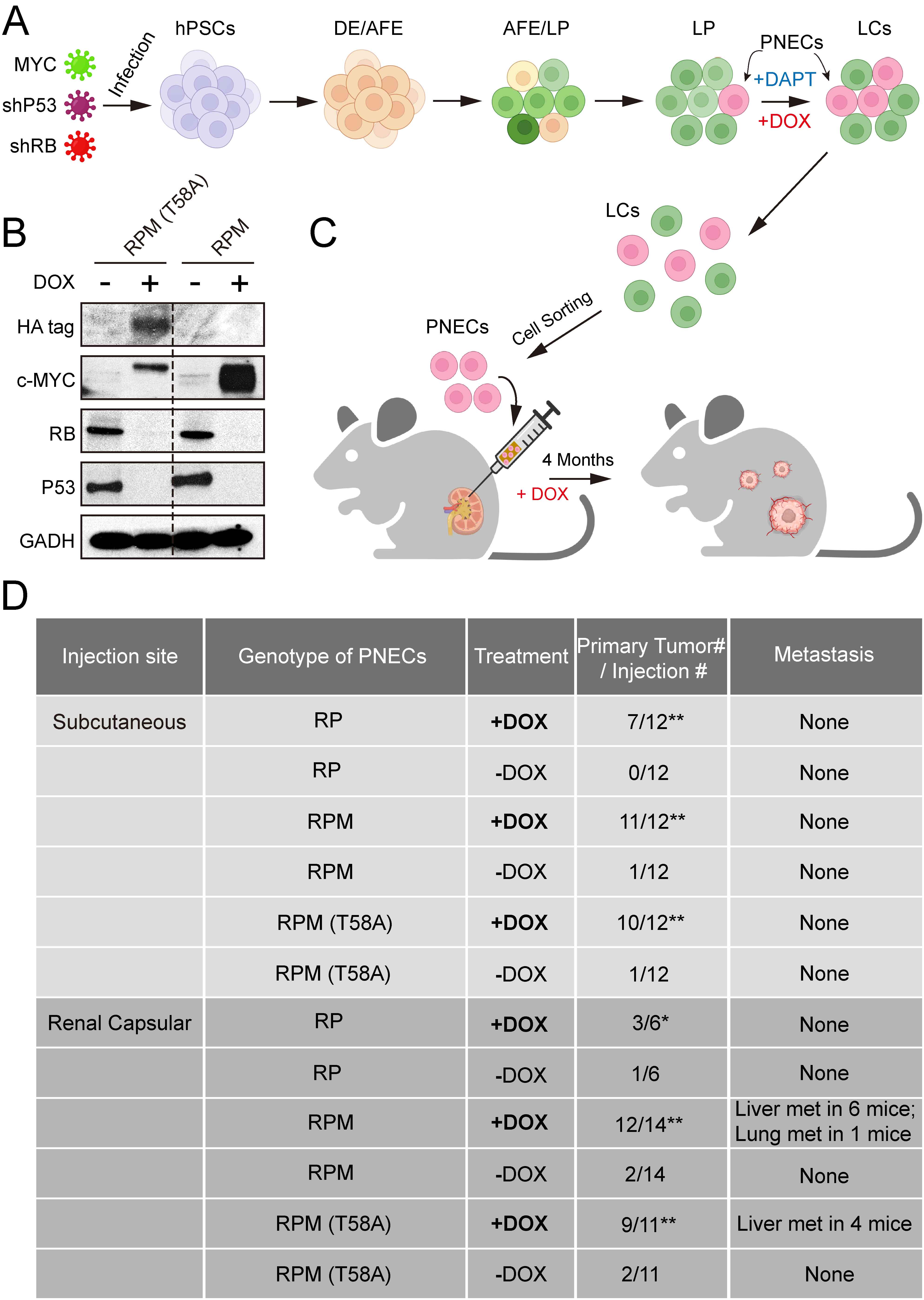

Supplement: Figure 1—source data 1. [file elife-93170-fig1-data1.zip › Figure 1-source data/Figure 1.jpg]

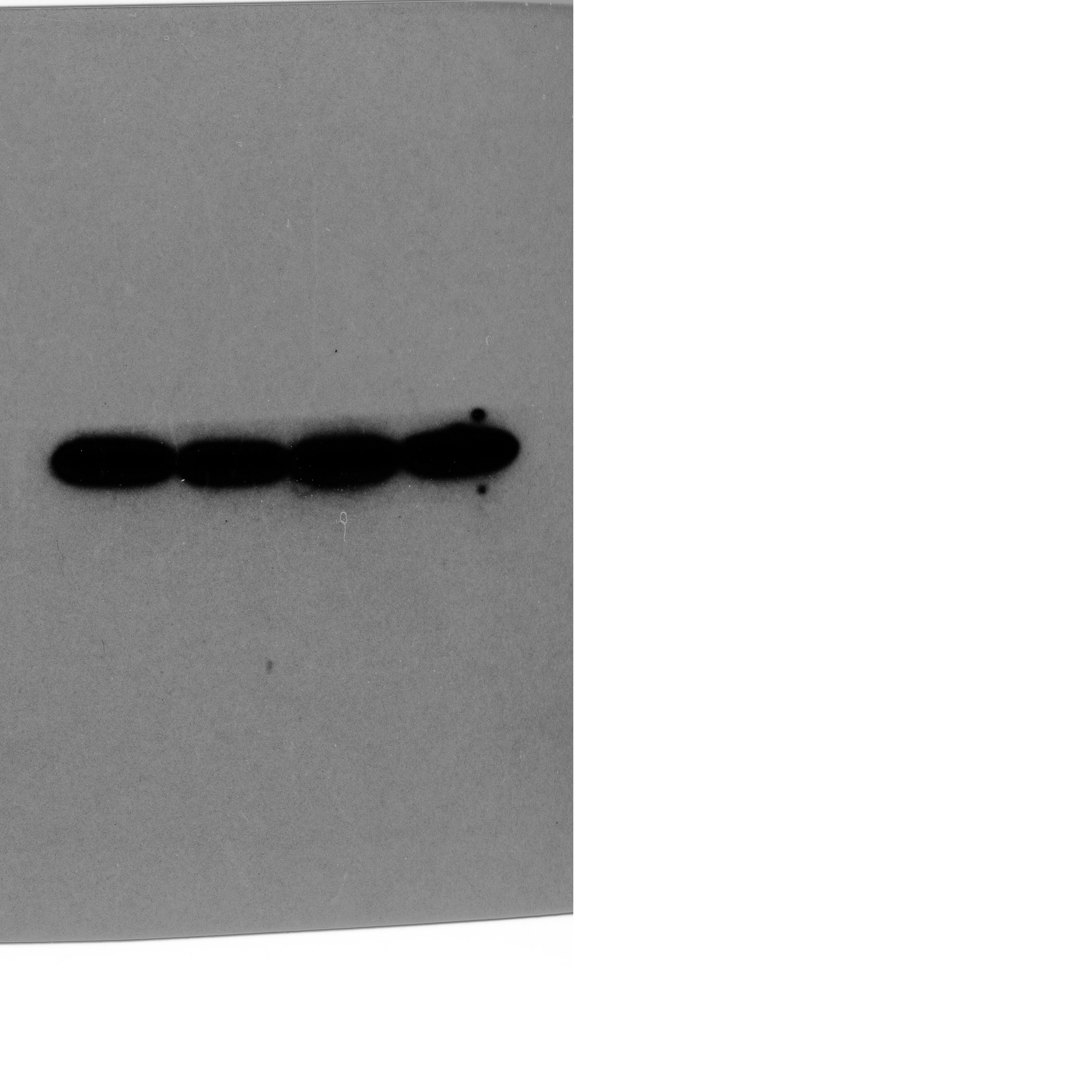

Supplement: Figure 1—source data 1. [file elife-93170-fig1-data1.zip › Figure 1-source data/Raw data_Fig 1B_GAPDH.png]

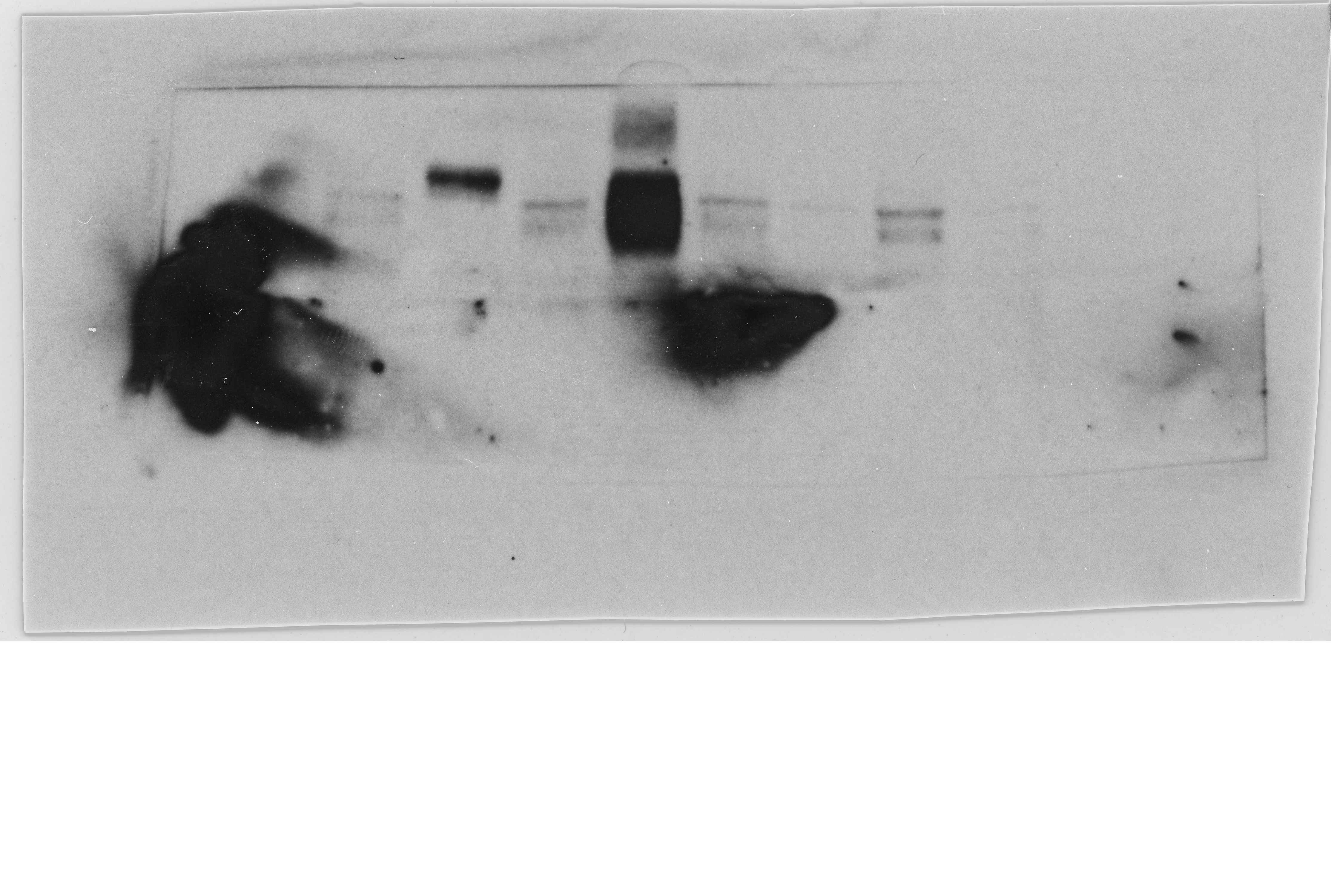

Supplement: Figure 1—source data 1. [file elife-93170-fig1-data1.zip › Figure 1-source data/Raw data_Fig 1B_MYC-Blot.png]

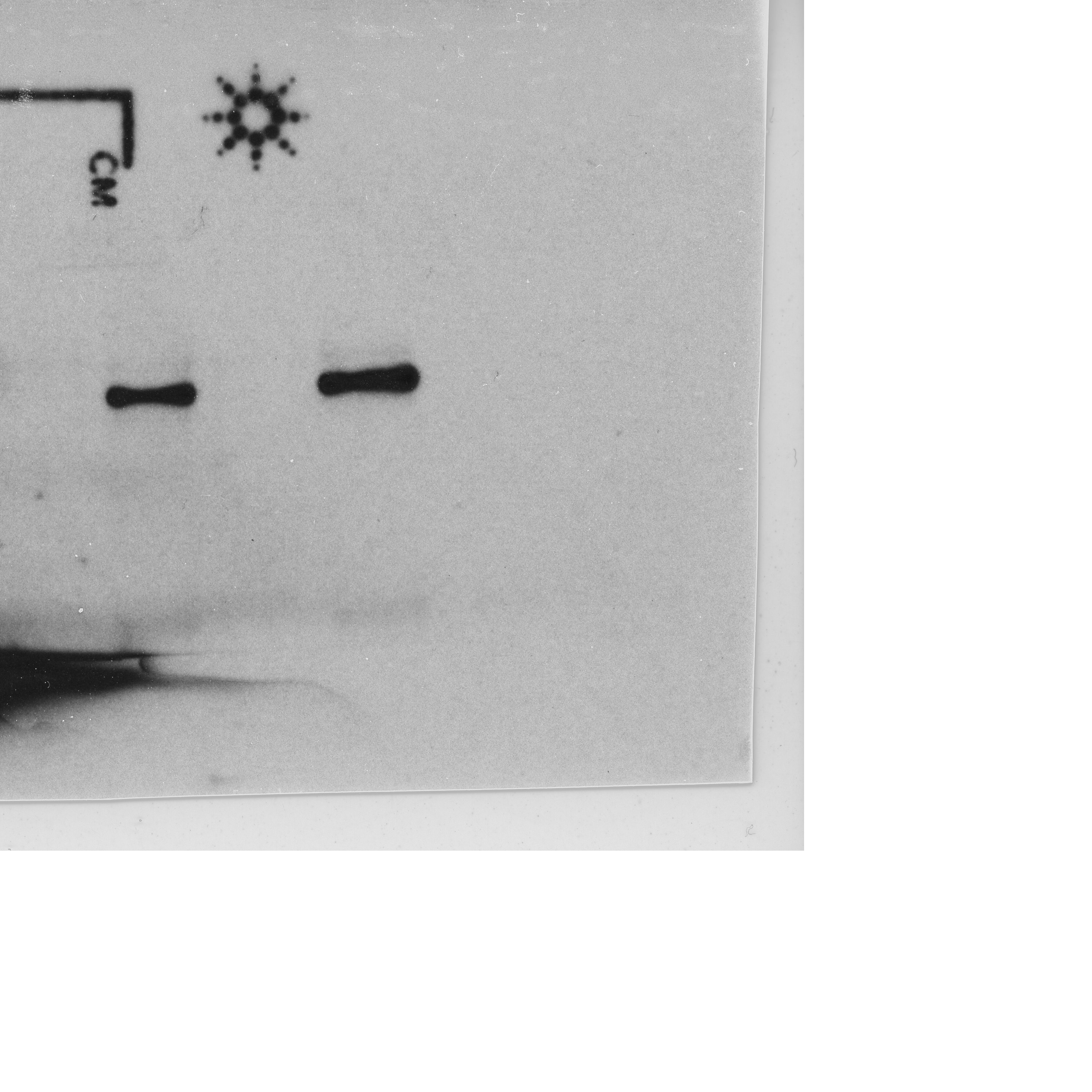

Supplement: Figure 1—source data 1. [file elife-93170-fig1-data1.zip › Figure 1-source data/Raw data_Fig 1B_P53.png]

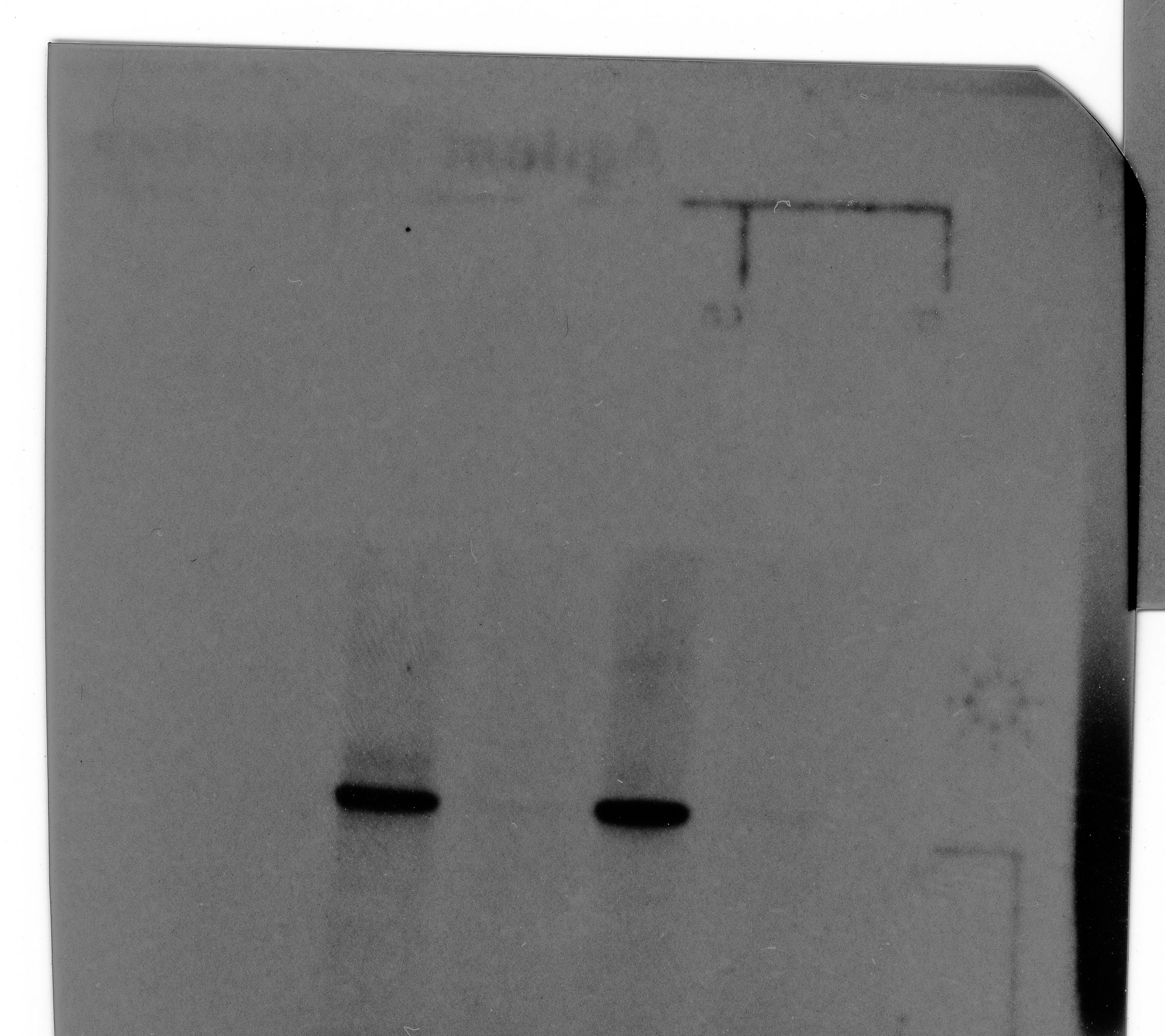

Supplement: Figure 1—source data 1. [file elife-93170-fig1-data1.zip › Figure 1-source data/Raw data_Fig 1B_RB.tif]

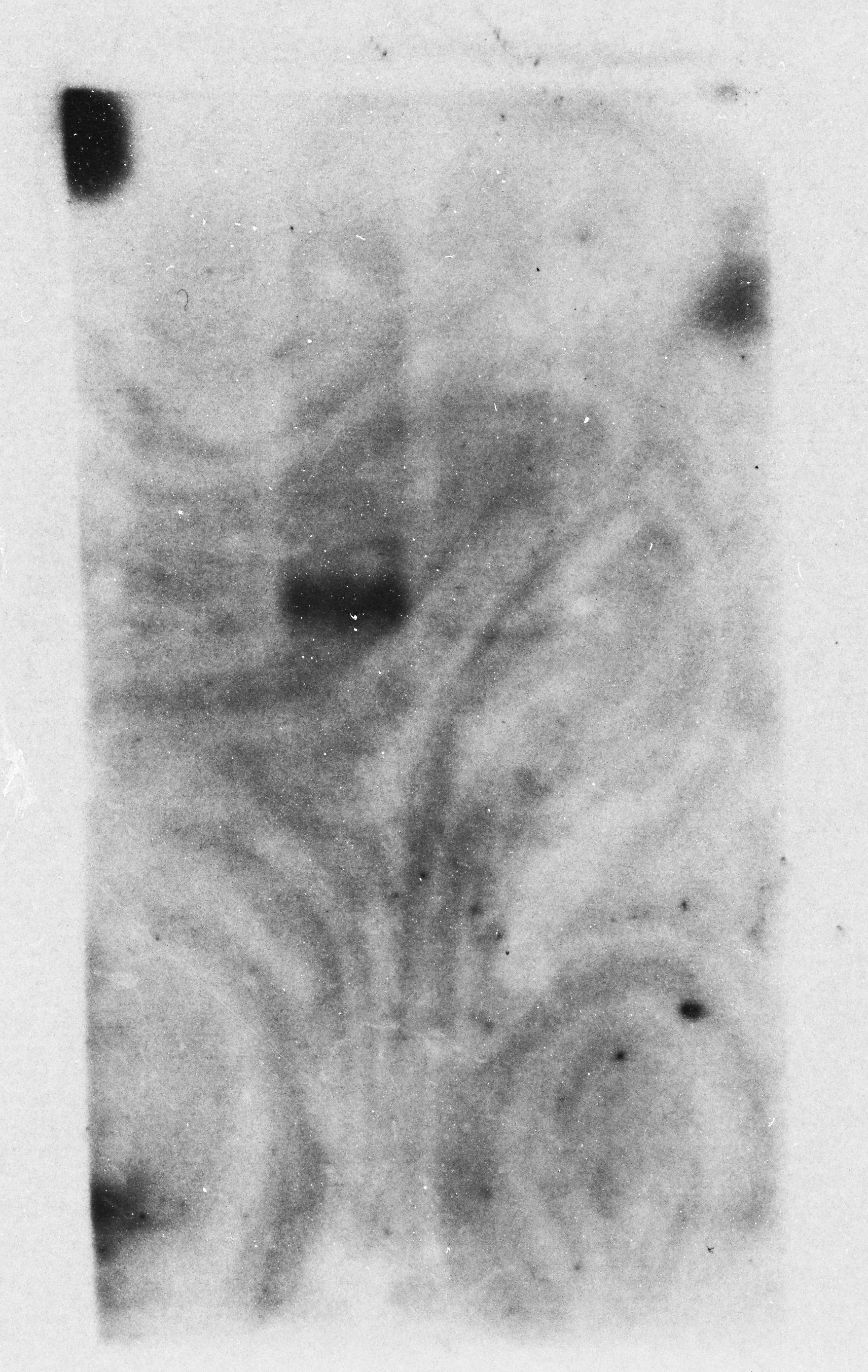

Supplement: Figure 1—source data 1. [file elife-93170-fig1-data1.zip › Figure 1-source data/Raw dta_Fig 1B_HA.tif]

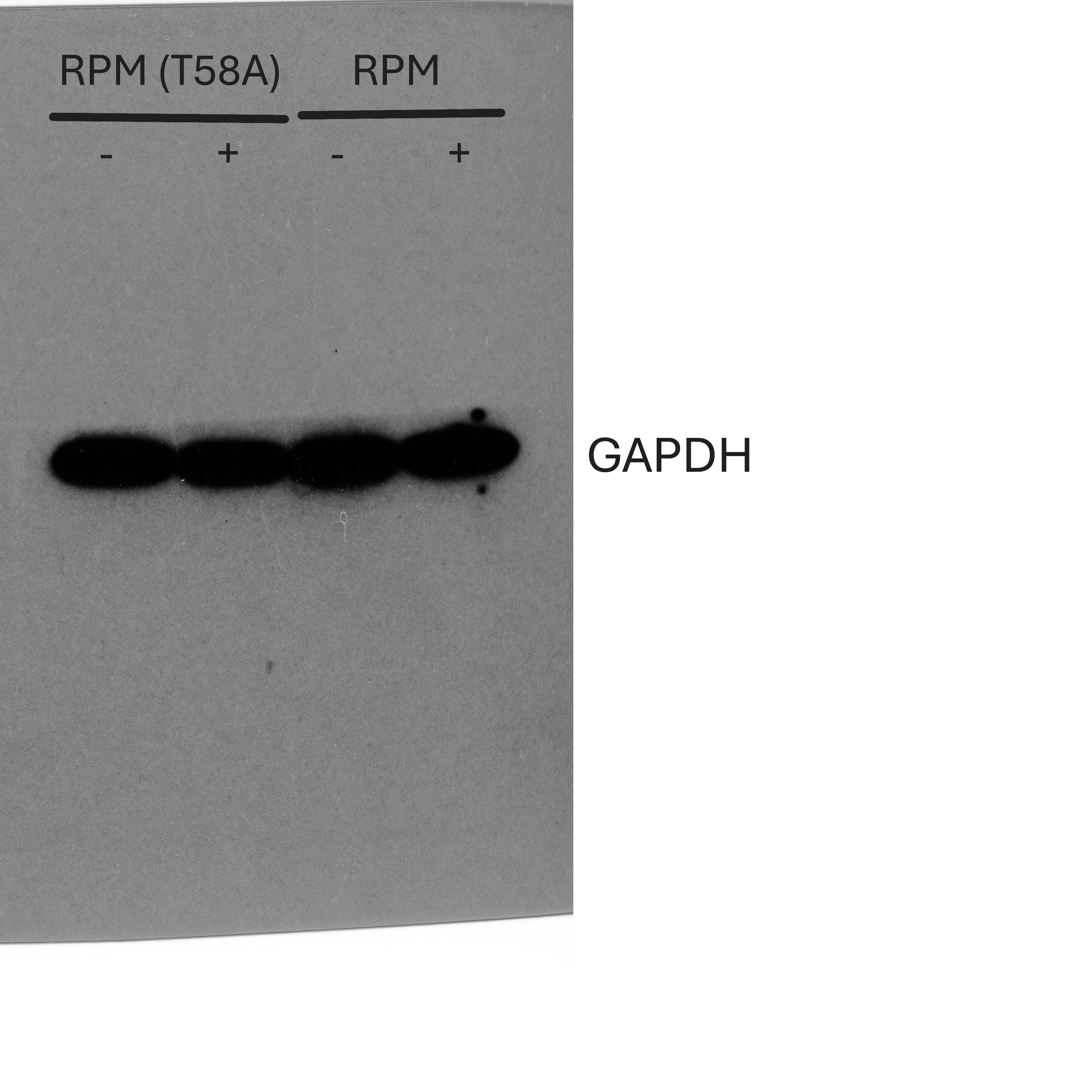

Supplement: Figure 1—source data 2. [file elife-93170-fig1-data2.zip › Figure 1-source data_labelled WB bands/Labelled_Raw data_Fig 1B_GAPDH.JPG]

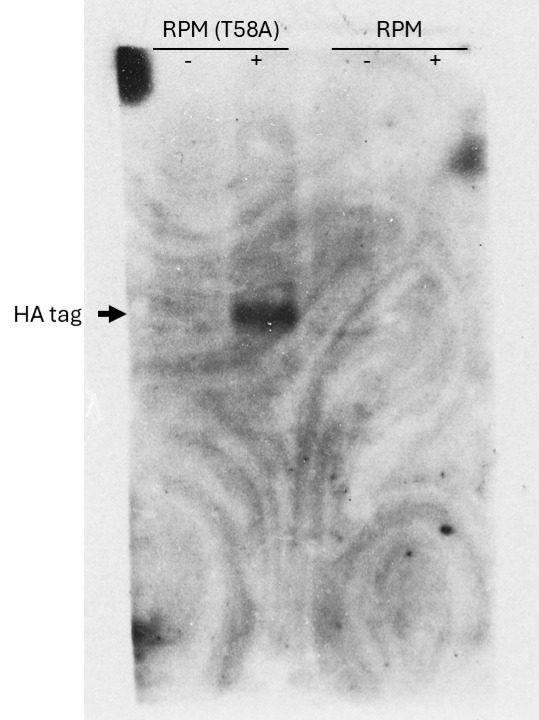

Supplement: Figure 1—source data 2. [file elife-93170-fig1-data2.zip › Figure 1-source data_labelled WB bands/Labelled_Raw data_Fig 1B_HA Tag.JPG]

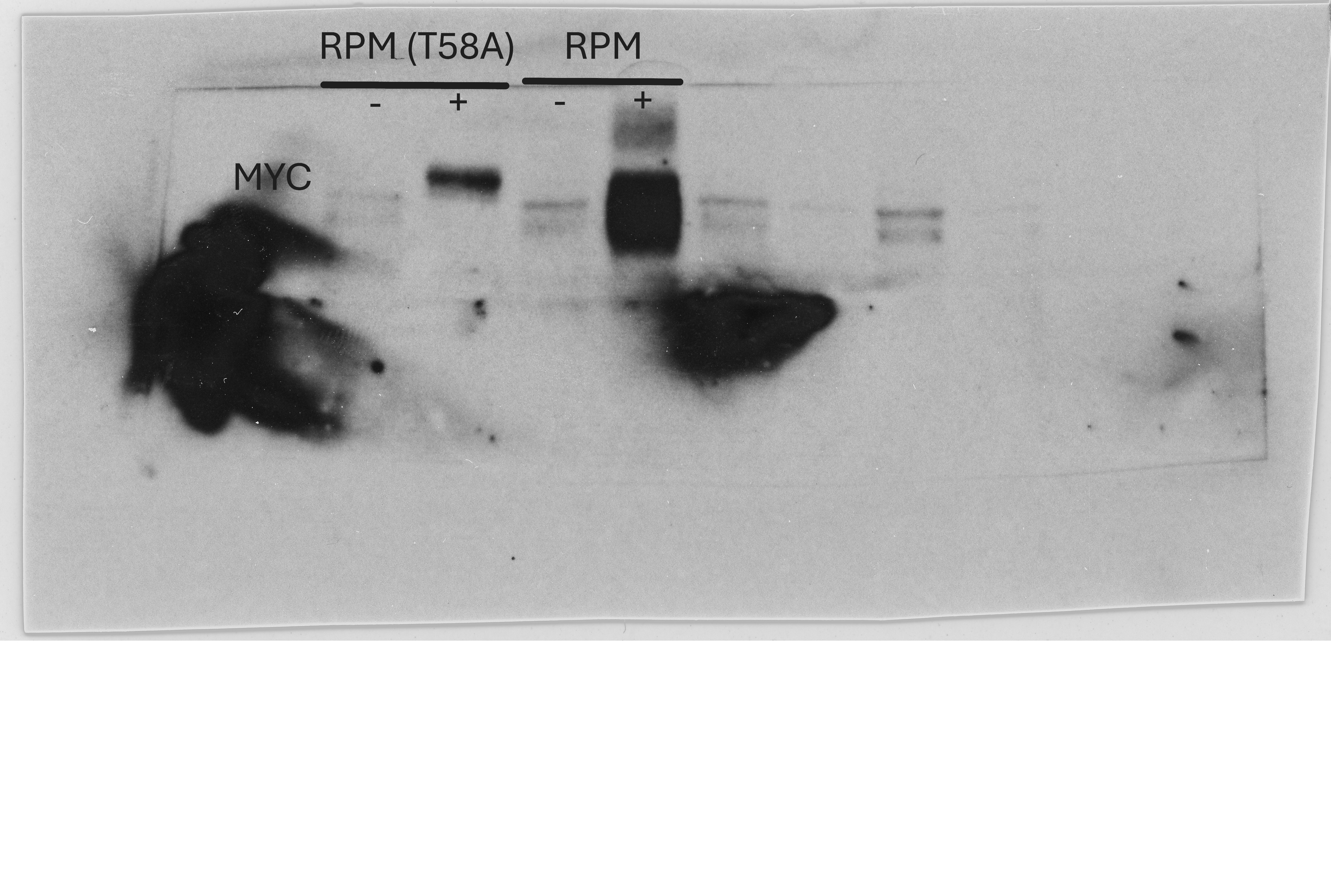

Supplement: Figure 1—source data 2. [file elife-93170-fig1-data2.zip › Figure 1-source data_labelled WB bands/Labelled_Raw data_Fig 1B_MYC-Blot.JPG]

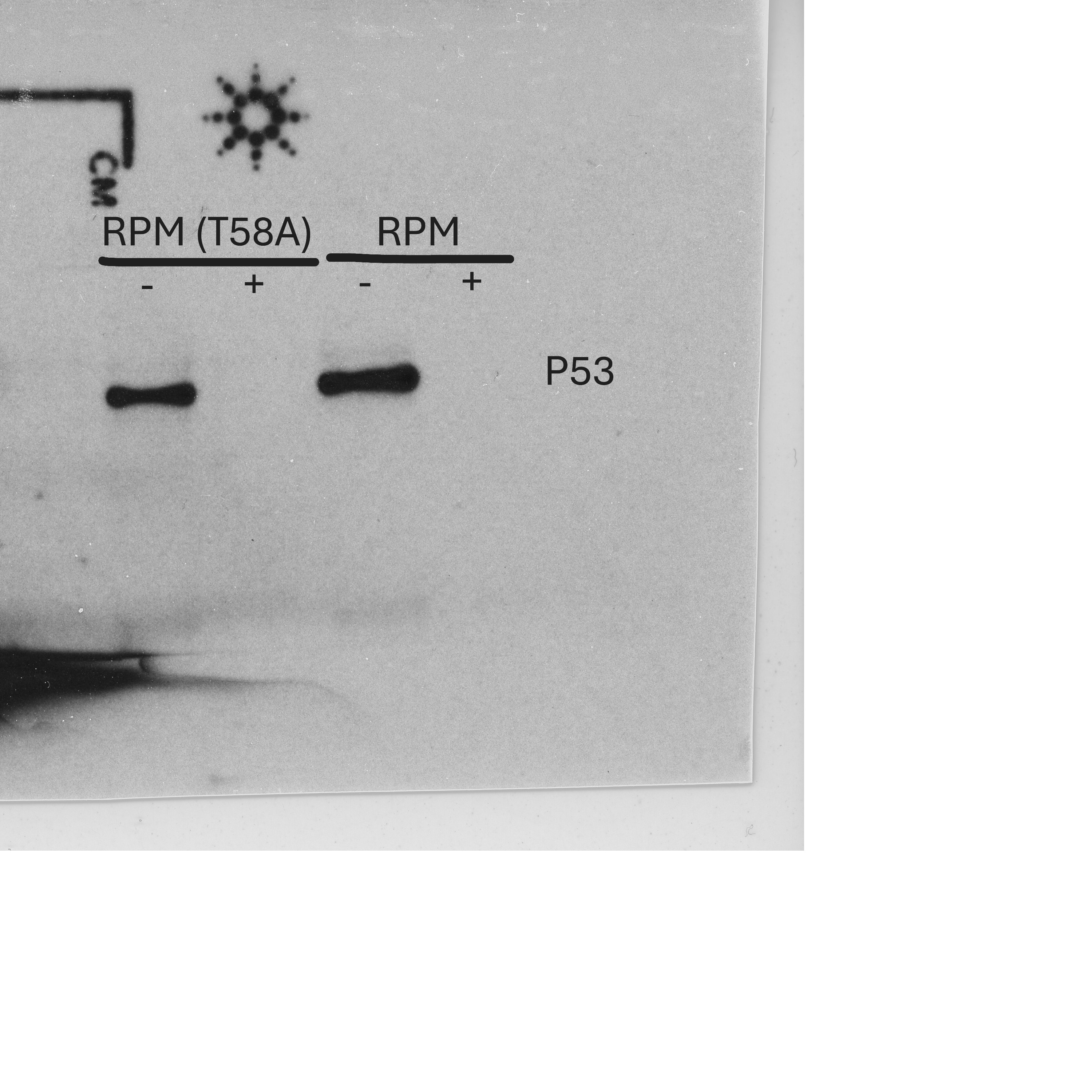

Supplement: Figure 1—source data 2. [file elife-93170-fig1-data2.zip › Figure 1-source data_labelled WB bands/Labelled_Raw data_Fig 1B_P53.JPG]

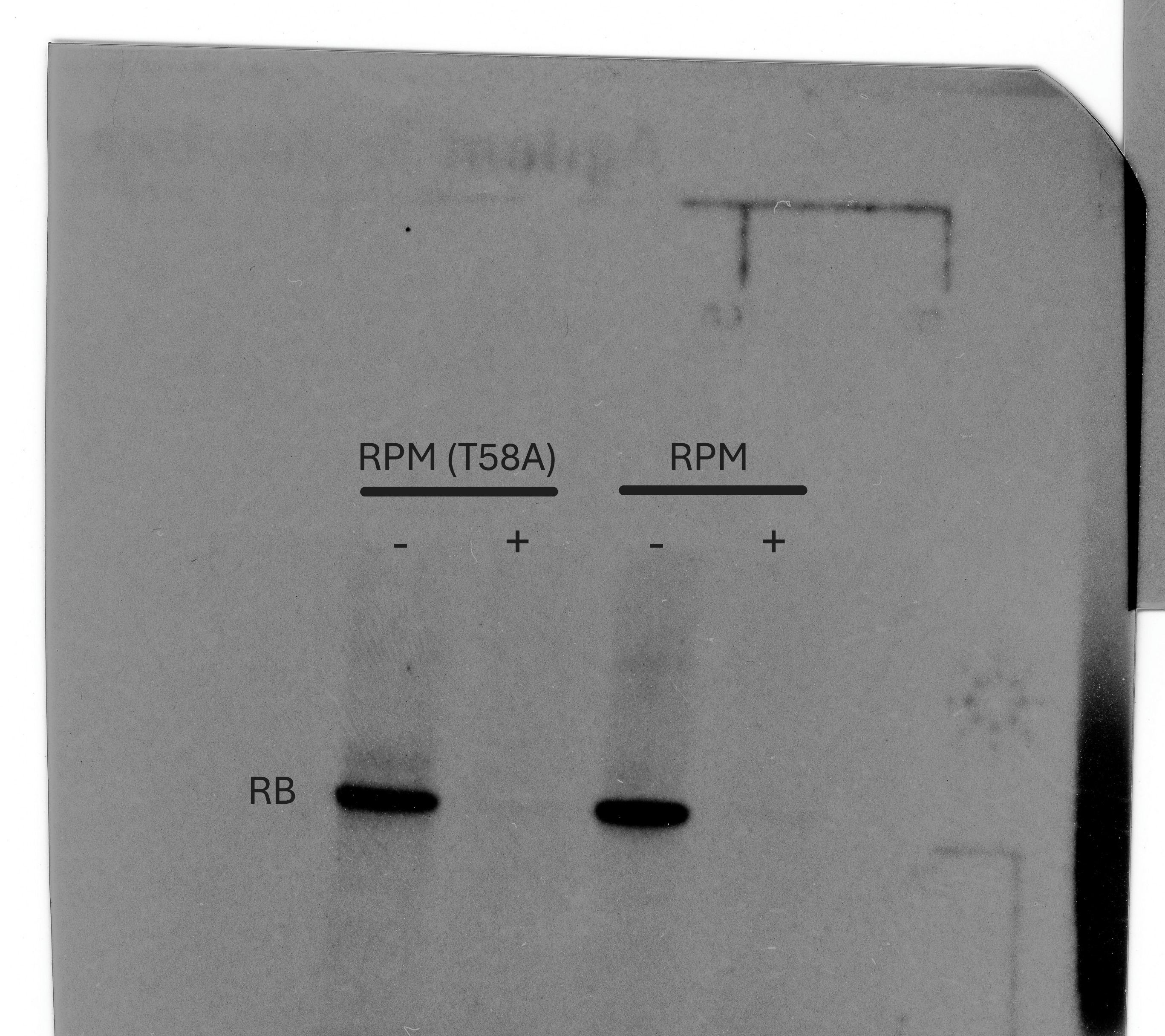

Supplement: Figure 1—source data 2. [file elife-93170-fig1-data2.zip › Figure 1-source data_labelled WB bands/Labelled_Raw data_Fig 1B_RB.JPG]

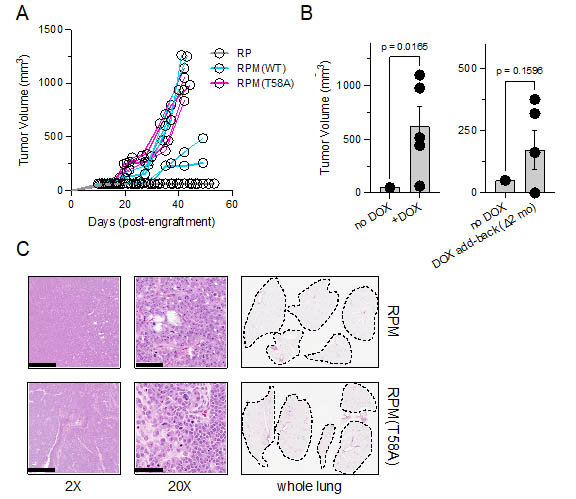

Supplement: Figure 2—source data 1. [file elife-93170-fig2-data1.zip › Figure 2-source data/Figure 2.jpg]

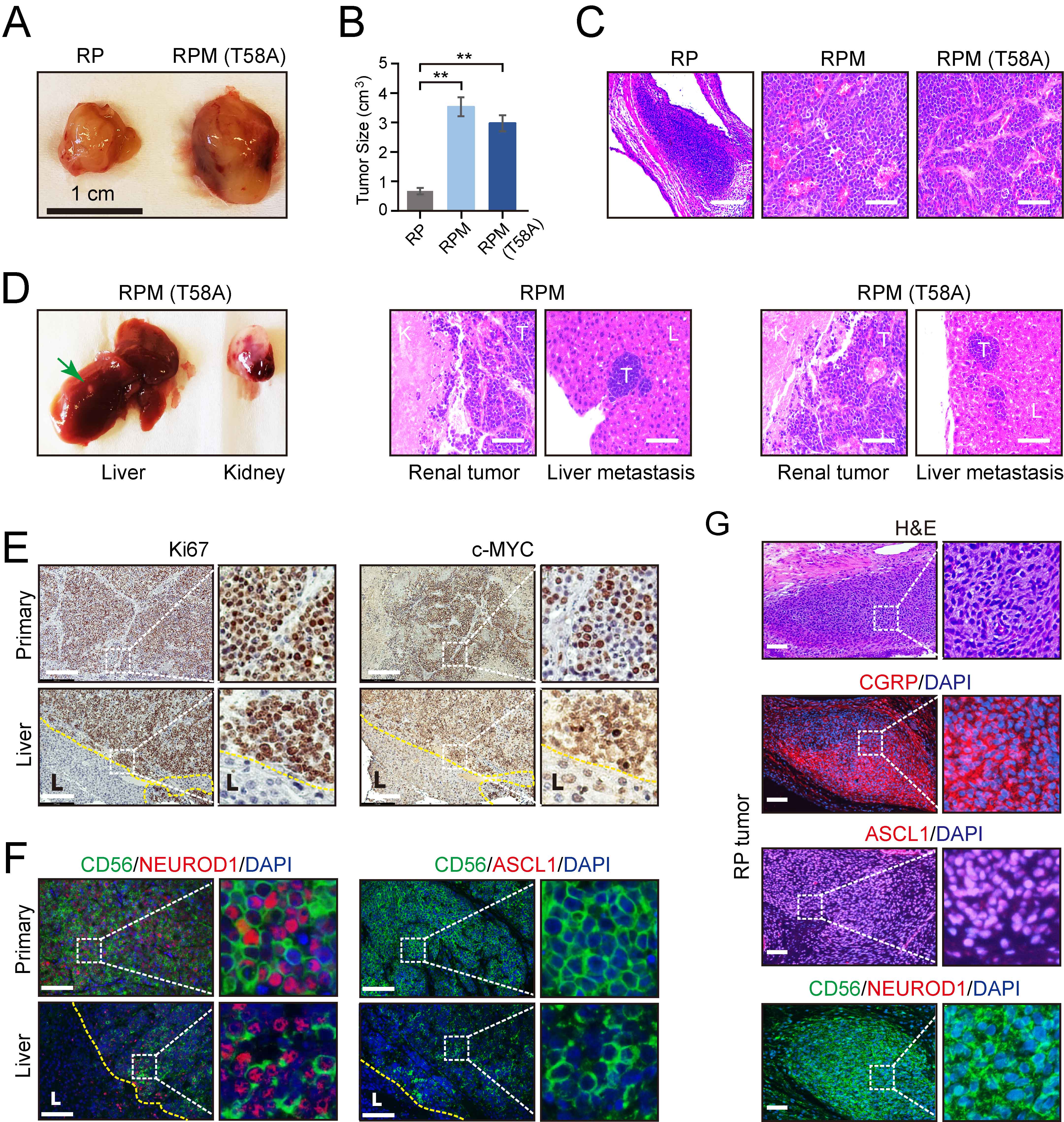

Supplement: Figure 3—source data 1. [file elife-93170-fig3-data1.zip › Figure 3-source data/Figure 3.jpg]

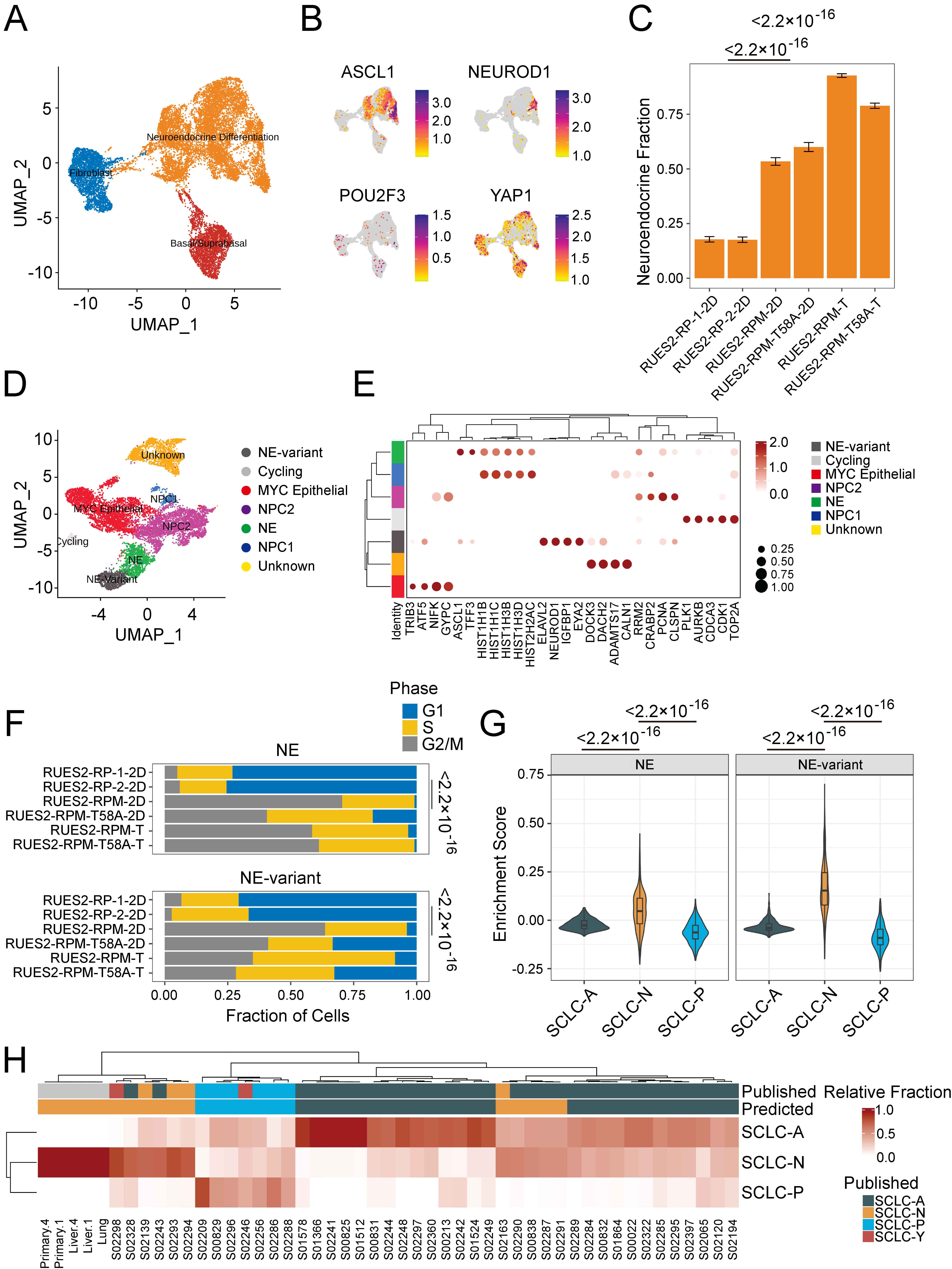

Supplement: Figure 4—source data 1. [file elife-93170-fig4-data1.zip › Figure 4-source data/Figure 4.jpg]

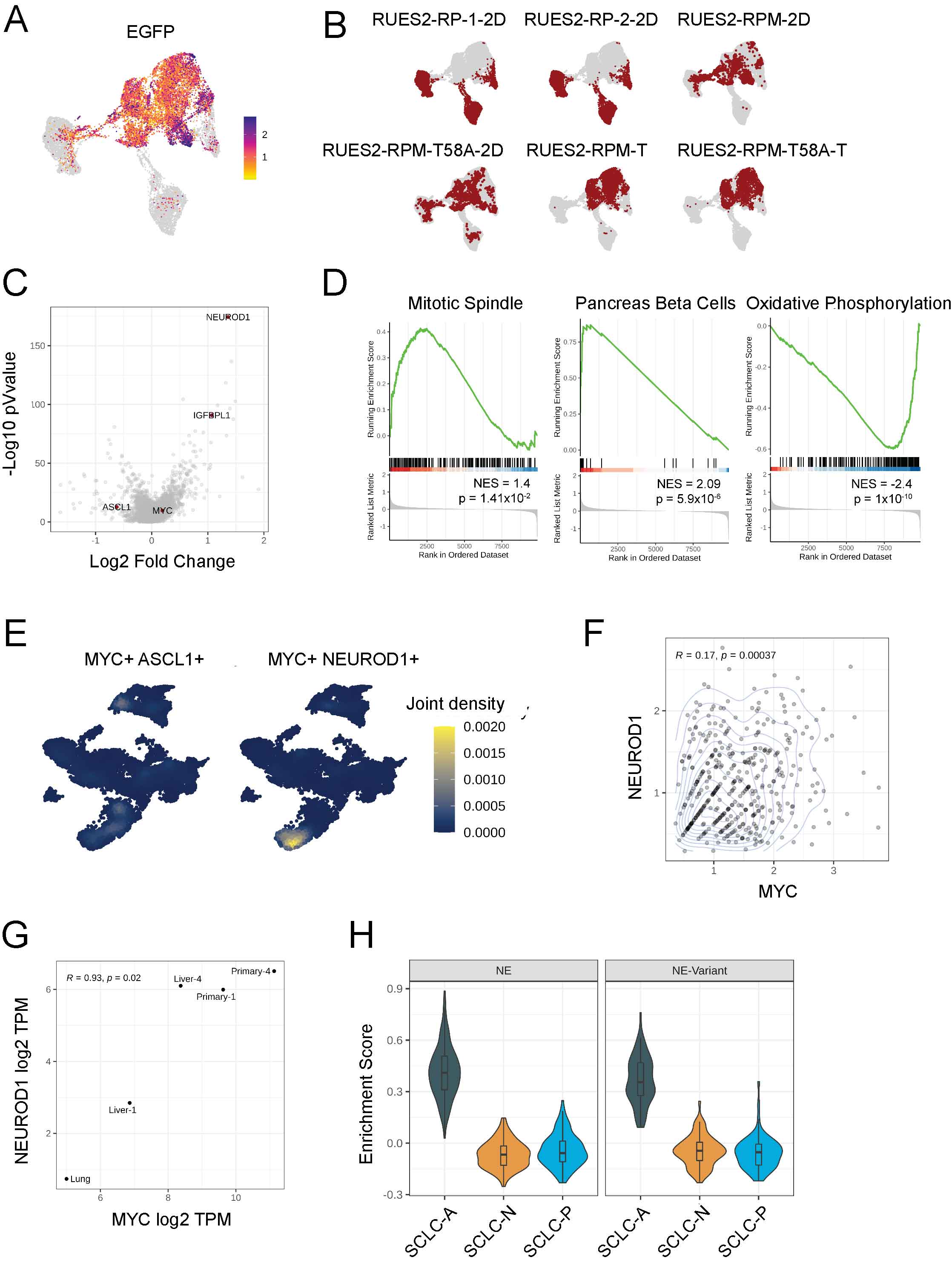

Supplement: Figure 4—figure supplement 1—source data 1. [file elife-93170-fig4-figsupp1-data1.zip › Supplement Fig 1-source data/Suppemental Figure S1.jpg]

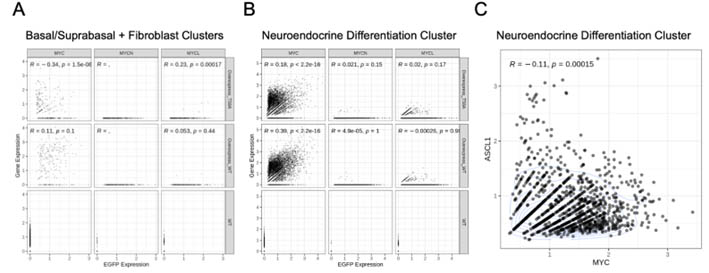

Supplement: Figure 4—figure supplement 2—source data 1. [file elife-93170-fig4-figsupp2-data1.zip › Supplement Fig 2-source data/Suppemental Figure S2.jpg]
