# Supplementary material for: Metastatic small cell lung cancer arises from TP53/RB1-deficient and MYC overproduction hESC-derived PNECs
Source: eLife. 2025 Jul 22;13:RP93170. doi: 10.7554/eLife.93170 (PMC12283072; doi:10.7554/eLife.93170)
Supplement: Source code 1. [file elife-93170-code1.zip › eLife-VOR-SR-2023-93170/Source Code File_270625.pdf]

```r
# ----------------------------------------------
# Single-cell RNA-seq pipeline: batch QC, integration, clustering, UMAP visualization
# ----------------------------------------------
library(Seurat)
library(harmony)
library(scds)
library(ggplot2)
library(dplyr)
library(Matrix)
library(patchwork)

# 0. PARAMETERS --------------------------------

# Root directory with all sample folders (each with 10x filtered_feature_bc_matrix/)
DATA_ROOT <- "data/scRNAseq"
# Sample metadata file (csv, at least two columns: sample_id, group)
META_FILE <- "data/sample_metadata.csv"
# Output folder for R objects and plots
OUT_DIR <- "results/scRNAseq_analysis"
dir.create(OUT_DIR, recursive = TRUE, showWarnings = FALSE)

# 1. LOAD SAMPLE METADATA ----------------------

metadata <- read.csv(META_FILE, stringsAsFactors = FALSE)
# Assume columns: sample_id, group
print(metadata)

# 2. BATCH READ ALL 10x SAMPLES ----------------

seurat.list <- list()
for (i in 1:nrow(metadata)) {
```

```
  sample_id <- metadata$sample_id[i]
  sample_group <- metadata$group[i]
  data_dir <- file.path(DATA_ROOT, sample_id, "filtered_feature_bc_matrix")
  cat("Reading", sample_id, "from", data_dir, "\n")

  counts <- Read10X(data.dir = data_dir)
  seu <- CreateSeuratObject(
    counts = counts,
    project = sample_id,
    min.cells = 3,
    min.features = 200
  )
  seu$orig.ident <- sample_id
  seu$group <- sample_group
  seurat.list[[sample_id]] <- seu
}
cat("Loaded", length(seurat.list), "samples.\n")

# 3. BASIC QC PER SAMPLE ------------------------

for (i in names(seurat.list)) {
  # Add percent.mt
  seu <- seurat.list[[i]]
  seu[["percent.mt"]] <- PercentageFeatureSet(seu, pattern = "^MT-")
  # QC plot for each sample
  VlnPlot(seu, features = c("nFeature_RNA", "nCount_RNA", "percent.mt"), ncol = 3)
  ggsave(file.path(OUT_DIR, paste0("QC_", i, ".png")), width = 10, height = 4)
  seurat.list[[i]] <- seu
}

# 4. MERGE SAMPLES & ADVANCED QC ----------------
```

```
# Merge all into one object, keep sample info
combined <- merge(seurat.list[[1]], y = seurat.list[-1], add.cell.ids = names(seurat.list), project = "Combined")

# Compute mitochondrial % for all
combined[["percent.mt"]] <- PercentageFeatureSet(combined, pattern = "^MT-")

# Visualize QC for all
VlnPlot(combined, features = c("nFeature_RNA", "nCount_RNA", "percent.mt"), group.by = "orig.ident", ncol = 3)
ggsave(file.path(OUT_DIR, "QC_all_samples.png"), width = 12, height = 5)

# Filter cells according to paper (adjust as needed)
combined <- subset(combined,
  subset = nFeature_RNA >= 1000 & nFeature_RNA <= 7500 &
         nCount_RNA >= 4000 & nCount_RNA <= 45000 &
         percent.mt < 10)

cat("After filtering, cell number:", ncol(combined), "\n")

# 5. REMOVE DOUBLETS ----------------------------

# Run scds doublet detection (hybrid method)
library(scds)
sce <- as.SingleCellExperiment(combined)
sce <- cxds_bcds_hybrid(sce)
doublet_scores <- colData(sce)$hybrid_score
# Here we use 95% quantile as doublet threshold (can tune)
threshold <- quantile(doublet_scores, 0.95)
is_doublet <- doublet_scores > threshold
combined <- combined[, !is_doublet]
cat("After doublet removal, cell number:", ncol(combined), "\n")
```

```
# 6. SCTransform, INTEGRATION (Harmony) ---------

combined <- SCTransform(combined, verbose = TRUE)
combined <- RunPCA(combined, verbose = TRUE)
# Batch integration by Harmony (across samples)
combined <- RunHarmony(combined, group.by.vars = "orig.ident", reduction = "pca", dims.use = 1:15,
max.iter.harmony = 50)

# 7. UMAP & CLUSTERING --------------------------
combined <- RunUMAP(combined, reduction = "harmony", dims = 1:15)
combined <- FindNeighbors(combined, reduction = "harmony", dims = 1:15)
combined <- FindClusters(combined, resolution = 0.1)
# Save Seurat object
saveRDS(combined, file = file.path(OUT_DIR, "combined_harmony_seurat.rds"))

# 8. BASIC VISUALIZATION ------------------------

# UMAP colored by sample
p1 <- DimPlot(combined, reduction = "umap", group.by = "orig.ident", label = TRUE) + ggtitle("UMAP by
Sample")
ggsave(file.path(OUT_DIR, "UMAP_by_sample.png"), p1, width = 8, height = 6)
# UMAP colored by group
p2 <- DimPlot(combined, reduction = "umap", group.by = "group", label = TRUE) + ggtitle("UMAP by Group")
ggsave(file.path(OUT_DIR, "UMAP_by_group.png"), p2, width = 8, height = 6)
# UMAP colored by cluster
p3 <- DimPlot(combined, reduction = "umap", label = TRUE) + ggtitle("UMAP by Cluster")
ggsave(file.path(OUT_DIR, "UMAP_by_cluster.png"), p3, width = 8, height = 6)

# Feature plots for key genes
genes_of_interest <- c("ASCL1", "NEUROD1", "POU2F3", "YAP1")
for (gene in genes_of_interest) {
```

```r
  if (gene %in% rownames(combined)) {
    p <- FeaturePlot(combined, features = gene, reduction = "umap")
    ggsave(file.path(OUT_DIR, paste0("FeaturePlot_", gene, ".png")), p, width = 7, height = 5)
  }
}

# 9. EXPORT CELL METADATA & CLUSTERING ----------
write.csv(combined@meta.data, file = file.path(OUT_DIR, "cell_metadata_all.csv"))

# 10. LOG SESSION INFO --------------------------
sink(file.path(OUT_DIR, "sessionInfo.txt"))
sessionInfo()
sink()
cat("Script finished successfully!\n")



# ----------------------------------------------
# scRNA-seq Analysis Part 2: Subtype scoring,
# cell type annotation, DE, enrichment, cell cycle, visualization
# ----------------------------------------------

library(Seurat)
library(clusterProfiler)
library(org.Hs.eg.db)
library(msigdbr)
library(enrichR)
library(ggplot2)
library(dplyr)
library(patchwork)

# 0. Load processed Seurat object -----------------------------
```

```r
SEURAT_FILE <- "results/scRNAseq_analysis/combined_harmony_seurat.rds"
OUT_DIR <- "results/scRNAseq_analysis/advanced"
dir.create(OUT_DIR, recursive = TRUE, showWarnings = FALSE)

seu <- readRDS(SEURAT_FILE)

# 1. Cell type annotation (manual/marker-based) --------
# If you have Azimuth or reference mapping, run here.
# Otherwise, use manual marker visualization to annotate clusters:
FeaturePlot(seu, features = c("ASCL1", "NEUROD1", "POU2F3", "YAP1"), reduction = "umap")
ggsave(file.path(OUT_DIR, "marker_featureplot.png"), width=9, height=5)
# Assign cluster identities (replace with real cluster IDs/markers as needed)
seu$celltype <- plyr::mapvalues(seu$seurat_clusters,
                         from = c(0,1,2),
                         to = c("NE", "Basal", "Fibroblast")) # Update as needed

DimPlot(seu, group.by="celltype", label=TRUE)
ggsave(file.path(OUT_DIR, "UMAP_celltype.png"), width=7, height=6)

# 2. Subtype module scoring (SCLC-A, SCLC-N, SCLC-P) ----------
# Prepare gene lists for each SCLC subtype (replace with your curated lists)
sclc_A_genes <- c("ASCL1", "INSM1", "SOX2")      # SCLC-A signature genes
sclc_N_genes <- c("NEUROD1", "GRIN2A", "GRIA2")  # SCLC-N signature genes
sclc_P_genes <- c("POU2F3", "SOX9")              # SCLC-P signature genes

seu <- AddModuleScore(seu, features=list(sclc_A_genes, sclc_N_genes, sclc_P_genes),
                  name=c("SCLCA", "SCLCN", "SCLCP"))

# Violin plots of module scores
VlnPlot(seu, features=c("SCLCA1","SCLCN1","SCLCP1"), group.by="celltype", pt.size=0)
ggsave(file.path(OUT_DIR, "SubtypeSignature_ViolinPlot.png"), width=9, height=6)
```

```
# 3. Marker gene discovery for each cell type -------------------
markers <- FindAllMarkers(seu, only.pos=TRUE, min.pct=0.25, logfc.threshold=0.25)
write.csv(markers, file.path(OUT_DIR, "all_markers_by_cluster.csv"))

top10 <- markers %>% group_by(cluster) %>% top_n(n=10, wt=avg_log2FC)
DoHeatmap(seu, features=top10$gene) + NoLegend()
ggsave(file.path(OUT_DIR, "top10_markers_heatmap.png"), width=12, height=10)

# 4. Differential Expression (e.g., between NE and non-NE) -------
#compare NE (ASCL1+) vs NE-variant (NEUROD1+), assuming clusters 0 & 1
Idents(seu) <- "celltype"
deg <- FindMarkers(seu, ident.1="NE", ident.2="NE-variant", test.use="MAST")
write.csv(deg, file.path(OUT_DIR, "DEG_NE_vs_NEvariant.csv"))

# 5. Enrichment analysis (clusterProfiler, msigdbr) --------------
msig_hallmark <- msigdbr(species = "Homo sapiens", category = "H")
deg_up <- rownames(deg)[deg$avg_log2FC > 0 & deg$p_val_adj < 0.05]
enrich_H <- enricher(deg_up, TERM2GENE = msig_hallmark[,c("gs_name", "gene_symbol")])
dotplot(enrich_H) + ggtitle("Hallmark Pathway Enrichment (upregulated)")
ggsave(file.path(OUT_DIR, "GSEA_DEG_Upregulated.png"), width=9, height=5)

# 6. Cell cycle analysis -----------------------------------------
cc.genes <- Seurat::cc.genes.updated.2019
seu <- CellCycleScoring(seu, s.features = cc.genes$s.genes, g2m.features = cc.genes$g2m.genes)
table(seu$Phase, seu$celltype)
# Plot fraction of cells in each phase per celltype
cell_cycle_plot <- ggplot(as.data.frame(table(seu$celltype, seu$Phase)),
                          aes(x=Var1, y=Freq, fill=Var2)) +
              geom_bar(stat="identity", position="fill") +
              labs(x="Cell Type", y="Fraction", fill="Phase") +
```

```
        theme_classic()
ggsave(file.path(OUT_DIR, "cell_cycle_fraction_by_celltype.png"), cell_cycle_plot, width=8, height=6)

# 7. Correlation/co-expression visualization ----------------------
# MYC vs NEUROD1 expression (scatter plot)
if (all(c("MYC", "NEUROD1") %in% rownames(seu))) {
  expr <- FetchData(seu, vars=c("MYC","NEUROD1"))
  cor_val <- cor(expr$MYC, expr$NEUROD1, method="pearson")
  p <- ggplot(expr, aes(x=MYC, y=NEUROD1)) +
    geom_point(size=0.3, alpha=0.3) +
    geom_smooth(method="lm", color="red") +
    annotate("text", x=max(expr$MYC,na.rm=TRUE), y=min(expr$NEUROD1,na.rm=TRUE),
             label=paste0("r=", round(cor_val,2)), hjust=1, vjust=0) +
    theme_classic()
  ggsave(file.path(OUT_DIR, "MYC_vs_NEUROD1_scatter.png"), p, width=7, height=6)
}

# 8. Save Seurat object with new annotations ----------------------
saveRDS(seu, file=file.path(OUT_DIR, "seurat_with_annotations.rds"))

# 9. Session Info -----------------------------------------------
sink(file.path(OUT_DIR, "sessionInfo.txt"))
sessionInfo()
sink()

cat("Script completed successfully!\n")




# ------------------------------------------------------------
# Bulk RNA-seq Analysis: Differential Expression, SCLC Subtype Deconvolution
```

```
# ------------------------------------------------------------

library(tximport)
library(DESeq2)
library(ComplexHeatmap)
library(BayesPrism)
library(ggplot2)
library(dplyr)

# 0. PARAMETERS ------------------------------------------------

# Path to sample metadata
META_FILE <- "data/bulk_metadata.csv" # at least: sample_id, group
# Path to Salmon quant outputs or counts matrix for all samples
BULK_DIR <- "data/bulk_salmon"
# Output directory
OUT_DIR <- "results/bulkRNAseq_analysis"
dir.create(OUT_DIR, recursive=TRUE, showWarnings=FALSE)

# 1. LOAD BULK RNA-SEQ COUNTS ----------------------------------

# Example: using Salmon quantification
samples <- read.csv(META_FILE, stringsAsFactors=FALSE)
files <- file.path(BULK_DIR, samples$sample_id, "quant.sf")
names(files) <- samples$sample_id

# Tximport for gene-level abundance
txi <- tximport(files, type="salmon", txOut=TRUE)

# 2. DIFFERENTIAL EXPRESSION ANALYSIS (DESeq2) -----------------
```

```r
dds <- DESeqDataSetFromTximport(txi, colData=samples, design=~group)
dds <- DESeq(dds)
res <- results(dds, contrast=c("group","Tumor","Normal"))
res <- lfcShrink(dds, coef=2, res=res)
write.csv(as.data.frame(res), file=file.path(OUT_DIR, "bulk_DEG_Tumor_vs_Normal.csv"))

# Volcano plot
res$gene <- rownames(res)
res$signif <- ifelse(res$padj < 0.05 & abs(res$log2FoldChange) > 1, "yes", "no")
ggplot(res, aes(x=log2FoldChange, y=-log10(padj), color=signif)) +
  geom_point(alpha=0.7, size=1) +
  scale_color_manual(values=c("grey","red")) +
  theme_minimal() +
  labs(title="Tumor vs Normal DEG Volcano", x="log2FC", y="-log10(padj)")
ggsave(file.path(OUT_DIR, "DEG_volcano.png"), width=8, height=6)

# 3. PREPARE REFERENCE FOR DECONVOLUTION -----------------------

# Load single-cell reference counts and annotation (should match BayesPrism requirements)
# These could come from your Seurat object, or a public reference
sc.counts <- readRDS("data/sc_reference_counts.rds")   # gene x cell matrix
sc.anno <- read.csv("data/sc_reference_annotation.csv") # columns: cell, cell_type, subtype

# Cell-type labels: must correspond to SCLC subtypes (e.g., SCLC-A, SCLC-N, SCLC-P, NSCLC, etc.)
cell_types <- sc.anno$subtype
names(cell_types) <- sc.anno$cell

# Aggregate counts per cell type for BayesPrism
bulk_mat <- as.matrix(txi$counts) # gene x sample

# 4. RUN BAYESPRISM FOR DECONVOLUTION -------------------------
```

```r
bp <- new("BayesPrism", sc.count.matrix=sc.counts,
          cell.type.labels=cell_types,
          bulk=bulk_mat)
bp <- run.prism(bp, outlier.cut=0.01, outlier.fraction=0.1)

# Extract and visualize fractions for SCLC subtypes
frac <- bp@cell.type.fraction
write.csv(frac, file=file.path(OUT_DIR, "SCLC_subtype_fractions.csv"))

# Heatmap for subtype composition
hm <- Heatmap(frac, name="Fraction", cluster_columns=TRUE, cluster_rows=TRUE,
             show_row_names=TRUE, show_column_names=TRUE)
pdf(file.path(OUT_DIR, "SCLC_subtype_fractions_heatmap.pdf"), width=8, height=5)
draw(hm)
dev.off()

# Barplot for SCLC subtypes per sample
frac_df <- as.data.frame(frac)
frac_df$subtype <- rownames(frac_df)
frac_long <- reshape2::melt(frac_df, id.vars="subtype", variable.name="sample", value.name="fraction")
ggplot(frac_long, aes(x=sample, y=fraction, fill=subtype)) +
  geom_bar(stat="identity", position="stack") +
  theme_minimal() +
  labs(title="SCLC Subtype Composition per Sample", y="Fraction", x="Sample")
ggsave(file.path(OUT_DIR, "SCLC_subtype_barplot.png"), width=9, height=6)

# 5. SAVE ALL SESSION INFO ------------------------------------
sink(file.path(OUT_DIR, "sessionInfo.txt"))
sessionInfo()
sink()
```

```
cat("Bulk RNA-seq and deconvolution analysis completed!\n")


# --------------------------------------------------------
# Figure Generation & Statistical Testing for Publication
# --------------------------------------------------------

library(Seurat)
library(ggplot2)
library(dplyr)
library(ComplexHeatmap)
library(cowplot)
library(reshape2)
library(ggpubr)

# 0. PARAMETERS & DATA -----------------------------------

# Paths to data from previous analyses
SEURAT_FILE   <- "results/scRNAseq_analysis/advanced/seurat_with_annotations.rds"
BULK_DEG_FILE <- "results/bulkRNAseq_analysis/bulk_DEG_Tumor_vs_Normal.csv"
DECONV_FILE   <- "results/bulkRNAseq_analysis/SCLC_subtype_fractions.csv"
OUT_DIR       <- "results/figures_for_publication"
dir.create(OUT_DIR, recursive=TRUE, showWarnings=FALSE)

# Load processed Seurat object
seu <- readRDS(SEURAT_FILE)
# Load DEG results (bulk RNA-seq)
bulk_deg <- read.csv(BULK_DEG_FILE, row.names=1)
# Load BayesPrism SCLC subtype fractions
deconv <- read.csv(DECONV_FILE, row.names=1, check.names=FALSE)
```

```
# 1. FIGURE: UMAP by cell type/cluster/sample -------------

p_umap_celltype <- DimPlot(seu, group.by="celltype", reduction="umap", label=TRUE) + ggtitle("Cell Type UMAP")
ggsave(file.path(OUT_DIR, "UMAP_by_celltype.png"), p_umap_celltype, width=8, height=7, dpi=300)

p_umap_sample <- DimPlot(seu, group.by="orig.ident", reduction="umap", label=FALSE) + ggtitle("Sample UMAP")
ggsave(file.path(OUT_DIR, "UMAP_by_sample.png"), p_umap_sample, width=8, height=7, dpi=300)

# 2. FIGURE: Violin plot of subtype scores ----------------

p_vln <- VlnPlot(seu, features=c("SCLCA1","SCLCN1","SCLCP1"), group.by="celltype", pt.size=0) +
  ggtitle("SCLC Subtype Signature Scores by Cell Type")
ggsave(file.path(OUT_DIR, "SubtypeScore_violin.png"), p_vln, width=10, height=7, dpi=300)

# 3. FIGURE: Heatmap of top marker genes -----------------

# Top markers for each cluster/celltype
markers <- read.csv("results/scRNAseq_analysis/advanced/all_markers_by_cluster.csv")
top_markers <- markers %>% group_by(cluster) %>% top_n(10, avg_log2FC)
hm <- DoHeatmap(seu, features=unique(top_markers$gene)) + NoLegend()
ggsave(file.path(OUT_DIR, "top_marker_heatmap.png"), hm, width=14, height=10, dpi=300)

# 4. FIGURE: Bulk RNA-seq volcano plot -------------------

bulk_deg$gene <- rownames(bulk_deg)
bulk_deg$signif <- ifelse(bulk_deg$padj < 0.05 & abs(bulk_deg$log2FoldChange) > 1, "yes", "no")
p_volcano <- ggplot(bulk_deg, aes(x=log2FoldChange, y=-log10(padj), color=signif)) +
  geom_point(alpha=0.7, size=1) +
  scale_color_manual(values=c("grey","red")) +
```

```
  theme_minimal() +
  labs(title="Bulk Tumor vs Normal DEG Volcano", x="log2FC", y="-log10(padj)")
ggsave(file.path(OUT_DIR, "Bulk_DEG_volcano.png"), p_volcano, width=9, height=7, dpi=300)

# 5. FIGURE: SCLC Subtype deconvolution heatmap ----------

hm2 <- Heatmap(as.matrix(deconv), name="Fraction", cluster_columns=TRUE, cluster_rows=TRUE,
              show_row_names=TRUE, show_column_names=TRUE)
pdf(file.path(OUT_DIR, "SCLC_subtype_fractions_heatmap.pdf"), width=8, height=6)
draw(hm2)
dev.off()

# 6. FIGURE: Barplot of SCLC subtype fractions -----------

deconv_long <- reshape2::melt(as.matrix(deconv), varnames=c("subtype","sample"), value.name="fraction")
p_bar <- ggplot(deconv_long, aes(x=sample, y=fraction, fill=subtype)) +
  geom_bar(stat="identity", position="stack") +
  theme_minimal() +
  labs(title="SCLC Subtype Composition per Sample", y="Fraction", x="Sample")
ggsave(file.path(OUT_DIR, "SCLC_subtype_fraction_barplot.png"), p_bar, width=12, height=7, dpi=300)

# 7. FIGURE: Correlation plot for co-expression ----------

if (all(c("MYC","NEUROD1") %in% rownames(seu))) {
  expr <- FetchData(seu, vars=c("MYC","NEUROD1"))
  cor_val <- cor(expr$MYC, expr$NEUROD1, use="pairwise.complete.obs")
  p_cor <- ggplot(expr, aes(x=MYC, y=NEUROD1)) +
    geom_point(size=0.4, alpha=0.4) +
    geom_smooth(method="lm", color="red") +
    annotate("text", x=max(expr$MYC,na.rm=TRUE), y=min(expr$NEUROD1,na.rm=TRUE),
           label=paste0("r=", round(cor_val,2)), hjust=1, vjust=0) +
```

```
  theme_classic() + labs(title="MYC vs NEUROD1 Expression")
 ggsave(file.path(OUT_DIR, "MYC_vs_NEUROD1_scatter.png"), p_cor, width=7, height=6, dpi=300)
}

# 8. FIGURE: Cell cycle phase fractions ------------------

cell_cycle_table <- as.data.frame(table(CellType=seu$celltype, Phase=seu$Phase))
p_cycle <- ggplot(cell_cycle_table, aes(x=CellType, y=Freq, fill=Phase)) +
 geom_bar(stat="identity", position="fill") +
 labs(y="Fraction", title="Cell Cycle Phase Fractions by Cell Type") +
 theme_minimal()
ggsave(file.path(OUT_DIR, "cell_cycle_phase_fraction.png"), p_cycle, width=10, height=6, dpi=300)

# 9. STATISTICS: t-tests, ANOVA, Fisher's test ------------

# Example: Compare SCLCN1 score between NE and NE-variant
scores <- FetchData(seu, c("celltype", "SCLCN1"))
ttest_result <- t.test(SCLCN1 ~ celltype, data=scores, subset=celltype %in% c("NE", "NE-variant"))
capture.output(ttest_result, file=file.path(OUT_DIR, "t_test_NE_vs_NEvariant_SCLCN1.txt"))

# ANOVA for SCLCN1 across all cell types
anova_result <- aov(SCLCN1 ~ celltype, data=scores)
capture.output(summary(anova_result), file=file.path(OUT_DIR, "ANOVA_SCLCN1_across_celltypes.txt"))

# Fisher's exact test: example, cluster by group
cluster_table <- table(seu$seurat_clusters, seu$group)
fisher_result <- fisher.test(cluster_table)
capture.output(fisher_result, file=file.path(OUT_DIR, "fisher_test_cluster_by_group.txt"))

# 10. EXPORT SUMMARY TABLES AND SESSION INFO ---------------
```

```
write.csv(cell_cycle_table, file=file.path(OUT_DIR, "cell_cycle_phase_counts.csv"))
sink(file.path(OUT_DIR, "sessionInfo.txt"))
sessionInfo()
sink()

cat("Figure generation and statistics complete!\n")
```
